# Impact of Cerium Oxide on the State and Hydrogenation Activity of Ruthenium Species Incorporated on Mesocellular Foam Silica

**DOI:** 10.3390/ma15144877

**Published:** 2022-07-13

**Authors:** Kalina Grzelak, Maciej Trejda, Jacek Gurgul

**Affiliations:** 1 Department of Heterogeneous Catalysis, Faculty of Chemistry, Adam Mickiewicz University in Poznań, Uniwersytetu Poznańskiego 8, 61-614 Poznań, Poland; tmaciej@amu.edu.pl; 2 Laboratory of Surfaces and Nanostructures, Jerzy Haber Institute of Catalysis and Surface Chemistry, Polish Academy of Sciences, Niezapominajek 8, 30-239 Kraków, Poland; jacek.gurgul@ikifp.edu.pl

**Keywords:** ruthenium species, bimetallic catalysts, hydrogenation, levulinic acid

## Abstract

Herein, the impact of cerium species loaded on mesoporous silica of MCF type on the state and catalytic activity of ruthenium species was studied. Up to 20 wt.% of cerium was incorporated on the silica surface, whereas the same 1 wt.% of Ru loading was applied. The samples prepared were examined by low temperature N_2_ adsorption/desorption, XRD, XRF, ICP-OES, XPS and H_2_ chemisorption. The catalytic activity of the materials obtained was investigated in the transformation of levulinic acid to γ-valerolactone. It was documented that the presence of Ce favored an increase in the dispersion of ruthenium species, which had a positive impact on the hydrogenation activity for up to 10 wt.% of Ce. Nevertheless, the highest cerium loading had a negative influence on the textural parameters of the support.

## 1. Introduction

Metal particle size has a dominant impact on the activity of heterogeneous catalysts. Gold had been considered inactive for a long period of time until Haruta [1] reported a high conversion in the low-temperature oxidation of hydrogen and CO over gold nanoparticles (<10 nm). Since that time, the effect of metal particle size on catalytic performance has been widely investigated [2]. The preparation method, metal precursor, synthesis conditions and support type are the most common factors used to control metal particle size [3,4,5]. Dispersion of metal particles may be greatly dependent on the support nature. Newman et al. [6] studied the effect of Ru particle size on various supports (mesoporous silica, active carbon and metal oxides) and obtained particle sizes ranging from 1.5 to 256 nm. In another work [7], some changes in the acid-base properties of the support for Ru particles led to diverse results in the selectivity and conversion of 2,5-hexanedione hydrogenation.

Two factors are essential for generating small supported particles: surface area and electronic properties of the support. Loading Ru on porous, large surface area supports, such as activated carbon, template mesoporous carbon or SBA-15 together with the use of a thermal method led to generation of fine metal particles on catalyst surfaces, which gave very high conversion in the hydrogenation of aromatic compounds [8]. While large surface area is achievable for inert oxides such as silica, it is not the case for metal oxides which, however, exhibit strong metal–support interactions. Many attempts have been made with Ru particles loaded on different metal oxides, i.e., Al_2_O_3_ [9,10], CeO_2_ [11], TiO_2_ [12,13] or Fe_2_O_3_ [14], resulting in the observation of strong metal–support interaction, manifested as enhanced activity in catalytic reactions. CeO_2_ is specifically interesting due to its oxygen storage capacity, which has been greatly acknowledged in the field of catalysis, i.e., reforming, water–gas shift reaction, oxidation, hydrogenation reactions and photocatalysis [15]. Gao et al. [11] have documented the interaction between RuO_2_ and ceria which served as the support. A Ru/CeO_2_ system was applied in the hydrogenation of ethyl levulinate to γ-valerolactone. Oxygen vacancies and increased surface area of ceria were proven to effectively disperse Ru species. Herein, we are concerned with ruthenium particles loaded on the mesoporous silica support (MCF) doped with ceria. Both effects inducing interaction with Ru, namely the porous structure of the support and doping with another metal, were considered in composing an efficient catalyst for hydrogenation reaction.

## 2. Materials and Methods

### 2.1. Materials

All chemicals and materials used were purchased from commercially available sources and used without further purification. Tetraethyl orthosilicate—TEOS (>99%), Pluronic P123, 1,3,5-trimethylbenzene (>98%), ammonium fluoride (>99.99%), cerium(III) nitrate hexahydrate (>99.999%), sodium borohydride (99%) and levulinic acid (>99%) were purchased from Sigma-Aldrich. HCl (35%) was procured from STAN LAB. Ruthenium trichloride hydrate (35–40% of Ru) was purchased from Acros Organics (Gell, Belgium).

### 2.2. Synthesis of the Catalysts

MCF mesoporous silica was prepared according to the procedure described in detail elsewhere [16]. Pluronic P123 (8 g) was dissolved in the hydrochloric acid solution (17.52 g of concentrated HCl with 282.5 g of H_2_O) and mixed for 1 h with 1,3,5-trimethylbenzene (12 g) and NH_4_F (0.0934 g). Then, TEOS (17.054 g) was added and the synthesis mixture was stirred at 40 °C for 20 h to be further moved to an oven at 100 °C for another 24 h. Filtered out white solid was calcined at 500 °C (8 h).

The support was impregnated (wetness impregnation) with aqueous solutions of cerium(III) nitrate hexahydrate using different concentrations in order to load 5, 10 and 20 wt.% of cerium. MCF (1 g) was wettened with approximately 10 mL of water with a previously dissolved specific amount of cerium precursor (5, 10 and 20 wt.% of Ce calculated per 1 g of MCF). Then, the solvent was removed with the use of a rotary evaporator. The solids were calcined at 500 °C for 5 h. The samples are denoted with xCe/MCF acronyms, where x stands for weight % loading of cerium.

Next, the as-obtained samples (5Ce/MCF, 10Ce/MCF and 20Ce/MCF) as well as pristine MCF were impregnated with ruthenium. In the typical procedure, a powder sample (~1 g) was dispersed in a specific amount of RuCl_3_ solution (4.6549 g mL^−1^, assumed Ru loading of 1 wt.%) diluted in 50 mL of water. Next, the aqueous solution of NaBH_4_ (4.1 mL, 0.25 M) serving as a reducing agent for Ru precursor was added dropwise and vigorously stirred for 30 min. The solid was filtered out, washed with water, placed in a ceramic tube and dried in vacuum at 80 °C for 4 h.

### 2.3. Characterization Techniques

The materials prepared were characterized using different analytical techniques: XRD, XRF, ICP-OES, N_2_ adsorption/desorption, XPS and H_2_-chemisorption.

#### 2.3.1. X-ray Diffraction (XRD)

XRD measurements were performed using a Bruker AXS D8 Advance diffractometer (Bruker, Karlsruhe, Germany) with Cu Kα radiation (λ = 0.154 nm) in the 2θ range of 25–80° and at a step of 0.05°·s^−1^.

#### 2.3.2. N_2_ Adsorption/Desorption

Low temperature N_2_ adsorption/desorption measurements were performed using the Micromeritics ASAP 2020 instrument (Norcross, GA, USA). The sample (ca. 100 mg) was outgassed at 300 °C under vacuum (<1.3 Pa) for 8 h. The surface area was calculated using the BET method. The pore volume and diameter were calculated according to Broekhoff–de Boer method with BJH Fass correction.

#### 2.3.3. ICP-OES

Cerium content (wt.%) was determined by ICP-OES, Spectro Blue TI (SPECTRO Analytical Instruments GmbH, Kleve, Germany).

#### 2.3.4. XRF

Ru content was estimated with the use of X-ray fluorescence spectrometry (ED-XRF Canberra Packard spectrometer, model 1510; excitation source: Am-241, Meriden, CT, USA). MoO_3_ (molybdenum oxide) served as the internal standard used to normalize the analysis results. Before the measurement, a sample was dried at 150 °C. The XRF spectra were registered in the high energy range for the K_α_-line of ruthenium and for the standard. The results were processed using the QXAS software package (IAEA Laboratories, Seibersdorf, Seibersdorf, Austria). 

#### 2.3.5. X-ray Photoelectron Spectroscopy (XPS)

The X-ray photoelectron spectroscopy (XPS) measurements for as-synthesized samples (without any additional pre-treatment) were carried out with a hemispherical analyzer (SES R4000, Gammadata Scienta, Uppsala, Sweden). The unmonochromatized AlKα (1486.6 eV) X-ray source with the anode operating at 12 kV and 15 mA current emission was applied to generate core excitation. The energy resolution of the system, measured as a full width at half maximum (FWHM) for the Ag 3d_5/2_ excitation line, was 0.9 eV (pass energy 100 eV). The spectrometer was calibrated according to ISO 15,472:2001. The base pressure in the analysis chamber was about 1 × 10^−10^ mbar and about 3 × 10^−9^ mbar during the experiment. The analyzed area of the sample was about 4 mm^2^ (5 mm × 0.8 mm). All spectra were collected at the pass energy of 100 eV (with 25 meV step). Intensities were estimated by calculating the integral of each peak (CasaXPS 2.3.23), after subtraction of the Shirley-type background, and fitting the experimental curve with a combination of Gaussian and Lorentzian lines of variable proportions (70:30). The results are charge-corrected (C-C bond, 285.0 eV) because samples were weakly conductive.

#### 2.3.6. H_2_-Chemisorption

H_2_-chemisorption was conducted on an ASAP 2020C (Micromeritics, Norcross, GA, USA). Before the measurement a sample (ca. 0.2 g) was reduced at 350 °C (ramp rate 10 °C min^−1^) for 120 min. Then, the samples were evacuated for 1 h at the reduction temperature and cooled down to 100 °C. Hydrogen chemisorption isotherms were measured in the pressure range of 15–610 mmHg. Ru content used for calculations was extracted from XRF analysis.

### 2.4. Transformation of Levulinic Acid

The catalytic reaction was conducted in a 25 mL pressure batch Parr reactor. In a typical run, 20 mL of 0.5 M aqueous levulinic acid solution was mixed with 30 mg of a catalyst with no pretreatment. The actual mass of the sample was recalculated with respect to the water content. The reactor was flushed with helium and hydrogen three times. The reaction was carried out under 40 bar of hydrogen at 40 °C for 1 h and stirred vigorously (600 rpm). The only product (γ-valerolactone) was identified by GC-MS (Thermo Scientific, Waltham, MA, USA) equipped with a 30 m DB-1 column. The conversion and yield were quantified with the use of a GC (Thermo Scientific, Waltham, MA, USA) equipped with 30 m DB-1 column and a FID detector.

## 3. Results and Discussion

### 3.1. Textural Parameters of the Catalysts

The textural properties of the materials obtained were determined with the use of low temperature nitrogen physisorption. The results obtained are presented in Table 1 and Figure S1. The pristine silica material (MCF), which was used as a support for ceria and ruthenium species, exhibited a mesoporous structure as indicated by the characteristic adsorption/desorption isotherm presented in Figure S1. The isotherm observed is of type IVa according to IUPAC classification [17] and it is characterized by the presence of a hysteresis loop associated with capillary condensation and evaporation from the material’s pores with narrow size distribution. MCF materials consist of cells, formed due to the application of 1,3,5-trimethylbenzene in the synthesis, that are interconnected by windows [18]. Table 1 presents the parameters calculated from the adsorption/desorption isotherms. It can be noticed that the support shows a large specific surface area (726 m^2^ g^−1^), which can be beneficial for active phase immobilization. The estimated cell size is 23.9 nm, whereas the window size is 13.6 nm. The pristine MCF sample is also characterized by the relatively large pore volume (2.22 cm^3^ g^−1^).

The MCF support was first modified by impregnation with cerium species. This procedure did not have a negative impact on the material structure, as indicated by the N_2_ adsorption/desorption isotherms presented in Figure S1. The shape of the isotherms as well as of the hysteresis loops did not change much; however, the volume of nitrogen adsorbed clearly decreased. The data presented in Table 1 indicate that the modification with ceria leads to a systematic decrease in the surface area with increasing ceria loading. Nevertheless, the surface areas are still relatively large and range between 647 and 523 m^2^ g^−1^. The same is true for the pore volume, which is reduced by ca. half in the 20Ce/MCF sample. The subsequent impregnation of the cerium containing materials with ruthenium species led to a further decrease in textural parameters. As far as the changes in pore size are concerned, it should be noted that the impregnation of MCF with ruthenium species had a much greater impact on the size of windows that interconnect the cells. It suggests that impregnated species were localized very close to the windows, leading to a decrease in their size. The difference observed for samples containing different ceria loadings should be related to the size of ruthenium species and this issue will be discussed in the next paragraphs.

### 3.2. Efficiency of Metal Incorporation

The modification technique applied for preparation of ceria and ruthenium containing MCF materials, i.e., impregnation, in most cases allows obtaining the assumed loading of metals. To verify this statement, the concentration of Ce species in the bulk was estimated by the ICP method, and that of Ru species by XRF analysis. The results obtained are presented in Table 2. The assumed value of ruthenium species was reached for xCe/MCF materials. A little lower loading was measured for Ru/MCF. Satisfactory results were also obtained for ceria loading. Some small differences noted between assumed and obtained loadings could be related to the measurement error. Further, the concentration of both metals was also estimated on the basis of XPS data. Due to the method specification, the concentration of species was measured on the very thin layer of material surface only. For Ru/xCe/MCF materials, the concentration of ruthenium species was two times higher than in the bulk. In contrast, for the Ru/MCF sample, the amount of Ru estimated by XPS was just a little bit higher than that obtained by the XRF method. This phenomenon could be explained by the formation of much larger nanoparticles containing a part of Ru species inside so that they are not detected. This assumption was confirmed by the chemisorption analysis and will be described below. A similar observation as for Ru/MCF was made in relation to Ce for the ceria containing materials. The concentration of Ce species calculated from the XPS analysis was much lower than assumed. This could be also related both to the coverage of ceria by ruthenium species and to the large size of ceria crystals, which is supported by the size calculations on the basis of the XRD patterns, using the Scherrer equation, which will be presented in the next paragraph. However, the difference in ceria concentration in the bulk and on the material surface is very significant. Therefore, a lower amount of ceria measured by XPS than by ICP should be assigned to the location of ceria species inside the pores of MCF support.

### 3.3. Oxidation State of Metals

**Figure 1:**
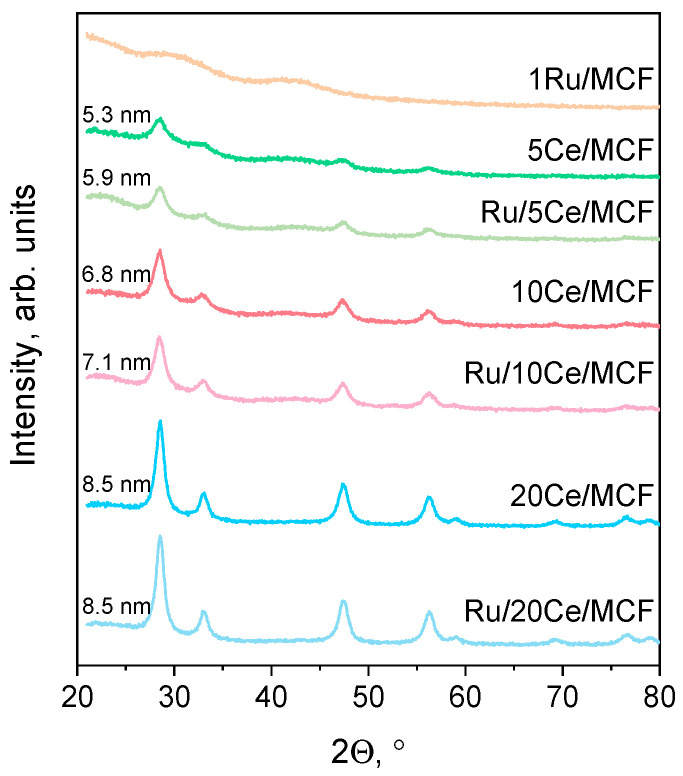
XRD patterns of the catalysts with the sizes of the ceria crystals calculated from the Scherrer equation.

The XRD technique was applied in order to identify the crystallographic forms of impregnated metals. The XRD patterns are presented in Figure 1. The reflexes characteristic of CeO_2_ [11] were observed for all Ce-modified samples; however, the width of peaks differed depending on the amount of ceria incorporated in the support. The sizes of the crystals formed were calculated from the Scherrer equation. The largest crystals were found in the Ru/20Ce/MCF sample, whereas the smallest ones (5.3 nm) were found in the 5 Ce/MCF material. It should be noted that the measured sizes of ceria particles make them possible to localize inside the pores of the MCF support. Moreover, no ruthenium species were detected either for Ru/Ce-modified samples or for the monometallic Ru/MCF catalyst. This result can suggest a strong amorphization of ruthenium and/or a good dispersion of ruthenium species.

XPS study provided a deeper insight into the forms of metals species on the catalysts’ surfaces and the results are shown in Table 3 and Figure S2. The Ru 3p_3/2_ region for all catalysts revealed two main components and one satellite. The contributions at BE of 461.7–461.9 eV and 463.8–464.4 eV can be assigned to the presence of Ru^0^ and oxidized ruthenium species, respectively [19,20,21]. The satellite component is derived from RuO_2_ final state screening, in line with the compelling argument presented by Kim et al. [22].

Wang et al. [23] assigned the bands at 463.4–463.6 eV to Ru^3+^, while those in the range 464.8–465.0 eV were assigned to oxide species RuO_x_. On the basis of the data from selected literature, Morgan [20] reported the average BE for RuO_2_ at 463.2 eV and RuCl_3_ at 463.9 eV. It was further compared with the experimental results, allowing the assignment of RuO_2_, RuCl_3_ and Ru(OH)_3_ to the binding energy of Ru 3p_3/2_ at 462.6, 464.1 and 464.1 eV, respectively. The signal at 462.7 eV was also assigned to RuO_2_ by Ernst and Sloof [24]. The above presented discrepancies made it difficult to clearly identify the ruthenium species in the range from 463.8 to 464.5 eV. Therefore, Ru was also analyzed in the 3 d region. The Ru 3 d signal strongly overlaps with C 1 s; thus, a numerical separation was necessary. For all the samples, two main components were distinguished: one assigned to the Ru^0^ (279.7–280.2 eV) and the other to RuO_2_ (280.8–281.0 eV) [20,25,26,27,28]. The relative intensities for Ru 3 d components were different from those for the 3p region. This effect was probably related to the less intense 3p peaks, as well as the difference in the sampling depth (∼4 nm for Ru 3p compared to ∼4.5 nm for Ru 3 d). It is worth noting that there is an additional contribution (Ru^0^ in Table 3) at BE of 278 eV in the Ru 3 d spectra of Ru/MCF and Ru/5Ce/MCF samples. We believe that this peak is caused by the differential charging of the supported Ru particles compared with the MCF support surface. A similar phenomenon was observed earlier for metallic ruthenium supported on MgO, and has been explained as a final state effect coming from the higher internal conductivity of supported Ru in comparison to the conductivity of dielectric MgO [25].

**Figure 2:**
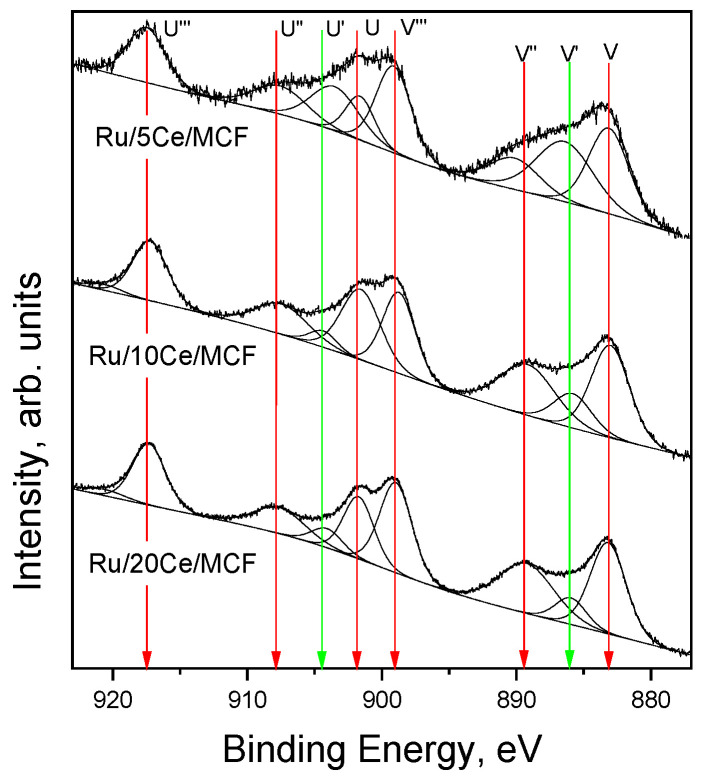
XPS spectra in Ce 3 d region.

The XPS results related to the Ce 3 d region are presented in Figure 2. The spectra are complex and were deconvoluted into eight components: two coming from the presence of Ce^3+^ (marked by green) and six others from Ce^4+^ (marked by red) [29,30]. The calculated Ce^3+^/(Ce^3+^ + Ce^4+^) ratio was much greater for Ru/5Ce/MCF (30.0%) than for Ru/20Ce/MCF (8.9%) and for Ru/10Ce/MCF (10.2%). Ru/5Ce/MCF, with the highest content of reduced cerium (Ce^3+^), also exhibited the highest content of metallic Ru (50.0%, Table 3) from among all the catalysts. Ruthenium content was extracted from XPS data and the obtained values were much higher than those from XRF analyses (Table 2), which indicates that Ru was present mainly on the surface of the catalysts.

### 3.4. H_2_ Chemisorption Analysis

For deeper characterization of the ruthenium species on the surface of MCF, the H_2_ chemisorption analyses were performed [31,32]. Based on these measurements, the ruthenium dispersion, particle size distribution and metal surface area were estimated. The results obtained are summarized in Table 4. The lowest dispersion of ruthenium species, ca. 2%, was found for the Ru/MCF sample. For this sample, the largest metal particle sizes were also detected, ca. 70 nm. This is in line with the measurements of Ru amount using XRF and XPS methods, showing similar values, which is not the case for Ru and Ce containing samples. The incorporation of ceria on the MCF surface allows a significant increase in the dispersion of Ru species, which for the lowest concentration of ceria reaches 6%. The further increase in ceria loading leads to an increase in Ru dispersion, which reaches ca. 16% the for Ru/20Ce/MCF sample. This finding is important because not only does the total amount of active species determine the catalytic activities of materials, but also their accessibility to the reactant. In the context of increased Ru dispersion, ceria should be considered a structural promoter. Having a low value of zero point charge, silica [33] in a water solution of RuCl_3_ is surrounded by a negative charge, opposite to ceria, which is positively charged. Therefore, ruthenium cations will interact with silica. The incorporation of ceria on the silica surface reduces the space accessible for ruthenium species. Moreover, the presence of ceria can protect against agglomeration during thermal treatment.

For the same Ru loading on xCe/MCF samples, with increasing ceria loading, the Ru specific surface area also increases and reaches the highest value of 0.636 m^2^ g^−1^ for Ru/20Ce/MCF. According to the literature, ceria can promote ruthenium particle dispersion [34,35]. An interesting observation was that the Ru particle size in xCe/MCF samples decreases with increasing ceria loading and is in the range between 8.1 and 22.1 nm. This indicates that these species can be localized to a higher extent inside the pores of the support.

### 3.5. Catalytic Testing in Hydrogenation of Levulinic Acid

The impact of ceria and its loading on the catalytic activity of ruthenium species was evaluated in a test reaction, i.e., liquid phase hydrogenation of levulinic acid into γ-valerolactone (GVL). To make the difference between materials tested more pronounced, the reaction was performed at a relatively low temperature of 40 °C.

**Figure 3:**
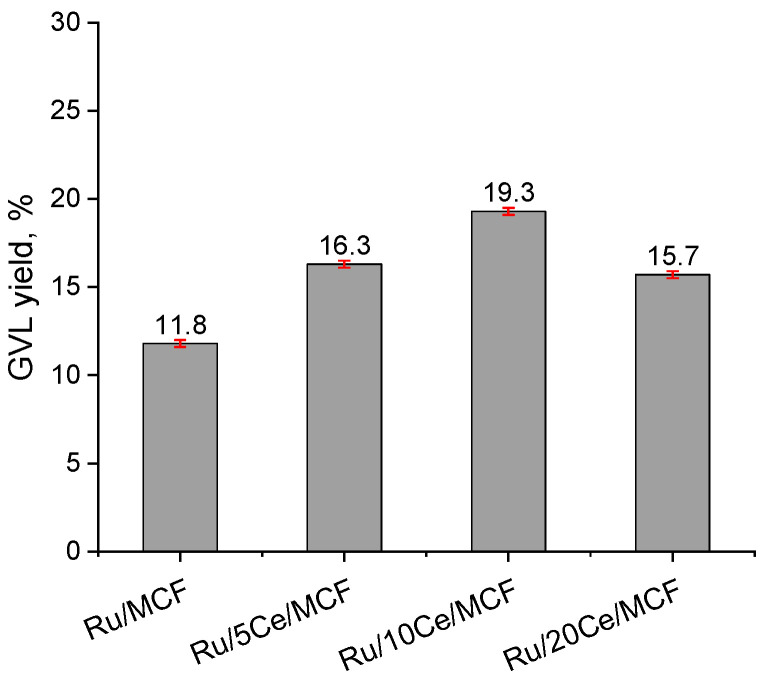
Catalytic activity of the catalysts in levulinic acid hydrogenation. GVL was the only identified product.

**Figure 4:**
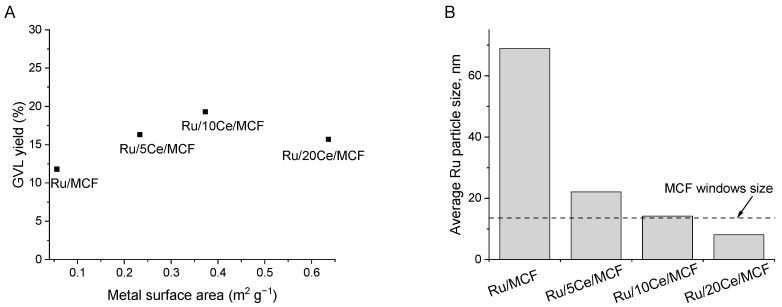
(**A**) The relation between GVL yield and metal surface area of Ru in the catalysts. (**B**) Average Ru particle size vs. MCF windows size.

For all the catalysts, GVL was observed as the only reaction product; thus, the results in Figure 3 are presented as the GVL yield. The lowest activity was observed for Ru/MCF, which is in line with the lowest metal dispersion. It was documented that with increasing ceria loading, the dispersion of the ruthenium species increases. The increase in dispersion of Ru species is accompanied by the increase in the activity of Ru/xCe/MCF samples up to 10 wt.% of ceria. The rate of a catalytic reaction should be proportional to the surface area of the active component, provided the reaction is not limited by mass transfer either within or outside the catalytic particles. The relation between the GVL yield and Ru metal surface area is presented in Figure 4A. For up to 10 wt.% of ceria, a very good linear correlation can be observed and the highest activity was observed for the Ru/10Ce/MCF sample. A further increase in ceria loading causes a significant decrease in the catalyst activity despite a relatively high dispersion. The estimated Ru particle size for this sample is ca. 8 nm, which makes the location of Ru species in the materials pores more possible (Figure 4B). Thus, the impact of diffusion effects should be considered. The incorporation of 20 wt.% of ceria causes a decrease in the pore volume as well as the size of interconnections between the cells, which obviously influence the migration of levulinic acid into the active species. Interestingly, diffusion limitations also explain the drop of activity in spite of the highest Ru metal surface area observed for this sample.

## 4. Conclusions

Mesostructured cellular foams with different loadings of ceria, up to 20 wt.%, were obtained and modified with the same amount of ruthenium species (1 wt.%). It was found that the presence of ceria on the silica support favors the increasing dispersion of Ru species and smaller particle size, which is beneficial for the hydrogenation properties of the catalysts obtained. The size of ruthenium species in the presence of a high ceria loading is small enough to permit their location inside the pores of the MCF support. It was concluded that as a result diffusion limitations cause such a catalyst to be less active in levulinic acid hydrogenation.

## Figures and Tables

**Table 1:** Textural parameters of the catalysts extracted from low temperature nitrogen physisorption measurements.

Catalyst	BET, m^2^ g^−1^	Pore Size ^a^, nm	Pore Size ^b^, nm	Pore Volume, cm^3^ g^−1^
MCF	726	23.9	13.6	2.22
Ru/MCF	565	23.8	13.7	1.64
5Ce/MCF	647	24.0	13.8	1.86
Ru/5Ce/MCF	445	22.3	13.5	1.25
10Ce/MCF	579	24.1	13.6	1.54
Ru/10Ce/MCF	441	21.7	11.8	1.37
20Ce/MCF	523	21.7	11.8	1.09
Ru/20Ce/MCF	542	22.0	7.9	1.16

^a^ Estimated from adsorption branches. ^b^ Estimated from desorption branches.

**Table 2:** Content of metals in the catalysts.

	Ru Content, wt.%			Ce Content, wt.%		
Catalyst	Assumed	XPS	XRF	Assumed	ICP	XPS
Ru/MCF	1.0	1.1	0.8	-	-	-
Ru/5Ce/MCF	1.0	2.3	1.1	5.0	3.5	2.2
Ru/10Ce/MCF	1.0	2.3	1.1	10.0	9.5	2.6
Ru/20Ce/MCF	1.0	2.3	1.1	20.0	20.8	3.5

**Table 3:** XPS results obtained from the Ru 3p_3/2_ and Ru 3 d_5/2_ regions.

BE, eV	Ru/MCF	Ru/5Ce/MCF	Ru/10Ce/MCF	Ru/20Ce/MCF
Ru 3p_3/2_, Ru^0^ Ru^4+^	461.7 (35%) 463.8 (65%)	461.9 (50%) 464.1 (50%)	461.9 (44%) 464.4 (56%)	461.7 (43%) 463.8 (57%)
Ru 3d_5/2_, Ru^0^	278.1 (24%)	277.9 (8%)		
Ru^0^	279.7 (31%)	280.2 (59%)	279.9 (46%)	280.0 (48%)
Ru^4+^	280.3 (45%)	281.0 (33%)	280.8 (55%)	281.0 (52%)

**Table 4:** Ruthenium dispersion in the presented samples.

Catalyst	Ru Dispersion, %	Ru Particle Size, nm	Ru Metal Surface Area, m^2^g ^−1^
Ru/MCF	1.93 ± 0.33	68.9 ± 11.7	0.056
Ru/5Ce/MCF	6.01 ± 1.04	22.1 ± 3.7	0.233
Ru/10Ce/MCF	9.37 ± 1.62	14.2 ± 2.4	0.373
Ru/20Ce/MCF	16.4 ± 2.8	8.1 ± 1.4	0.636

## Data Availability

The data presented in this study are available upon request from the corresponding author via e-mail: kalina.grzelak@amu.edu.pl.

## Supplementary Materials

The following supporting information can be downloaded at: https://www.mdpi.com/article/10.3390/ma15144877/s1, Figure S1: Isotherms of the catalysts, Figure S2: XP spectra in Ru 3p_3/2_ and Ru 3d region.
