# Supplementary material for: Impact of Cerium Oxide on the State and Hydrogenation Activity of Ruthenium Species Incorporated on Mesocellular Foam Silica
Source: Materials (Basel). 2022 Jul 13;15(14):4877. doi: 10.3390/ma15144877 (PMC9325213; doi:10.3390/ma15144877)
Supplement: Supplementary file 1 [file materials-15-04877-s001.zip › materials-1792918-supplementary.pdf]

Supplementary Information

Impact of cerium oxide on the state and hydrogenation activity of ruthenium species incorporated on mesocellular foam silica

Kalina Grzelak[1*], Maciej Trejda[1], Jacek Gurgul[2]

[1] Adam Mickiewicz University, Poznań, Faculty of Chemistry, Department of Heterogeneous Catalysis, Uniwersytetu Poznańskiego 8, 61-614 Poznań, Poland
[2] Polish Academy of Sciences, Jerzy Haber Institute of Catalysis and Surface Chemistry, Laboratory of Surfaces and Nanostructures, Niezapominajek 8, 30-239 Kraków, Poland

** corresponding author, e-mail: kalina.grzelak@amu.edu.pl*

List of content:

Figure S1. Isotherms of the catalysts.
Figure S2. XP spectra in Ru $3p_{3/2}$ and Ru 3d region.

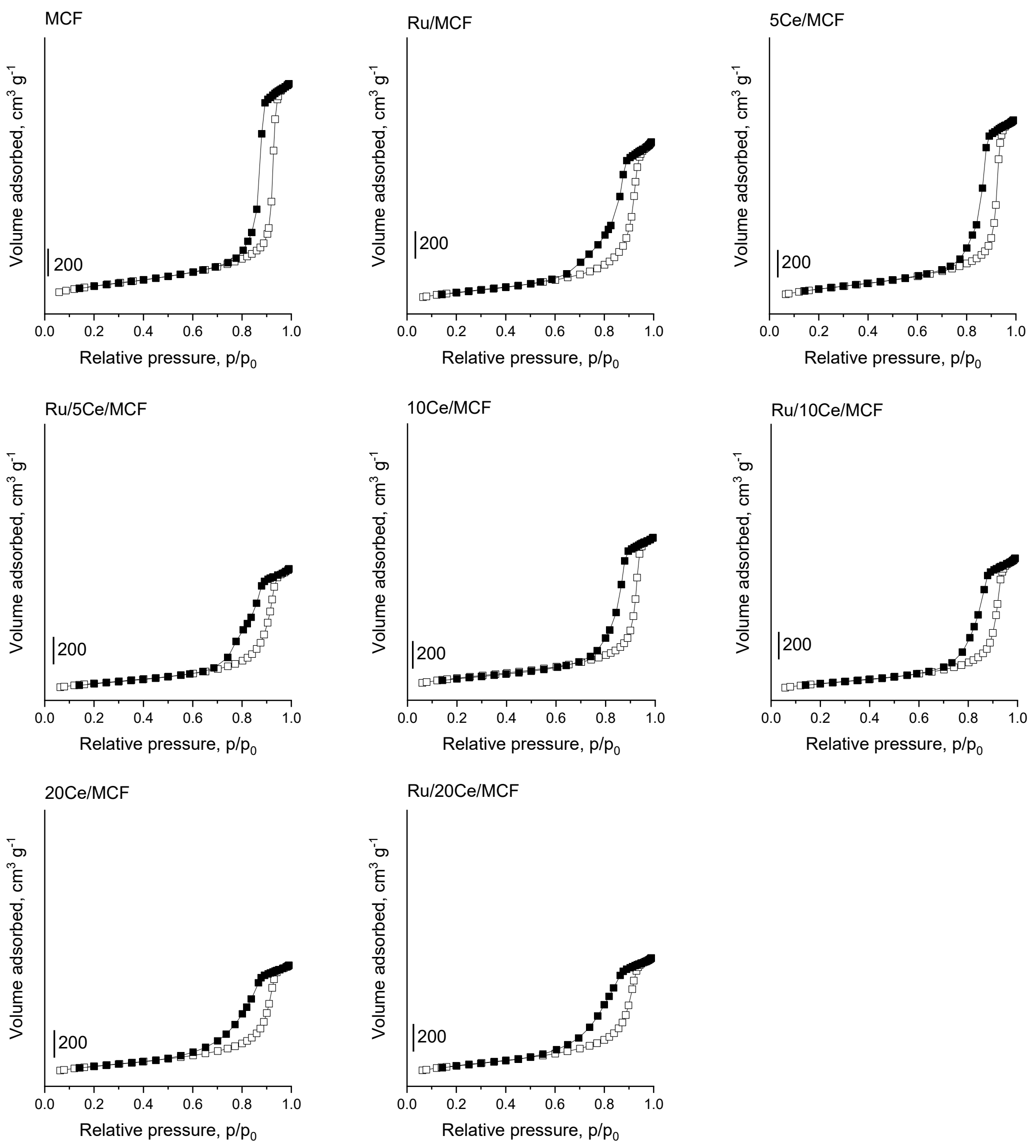

Figure S1. Isotherms of the catalysts.

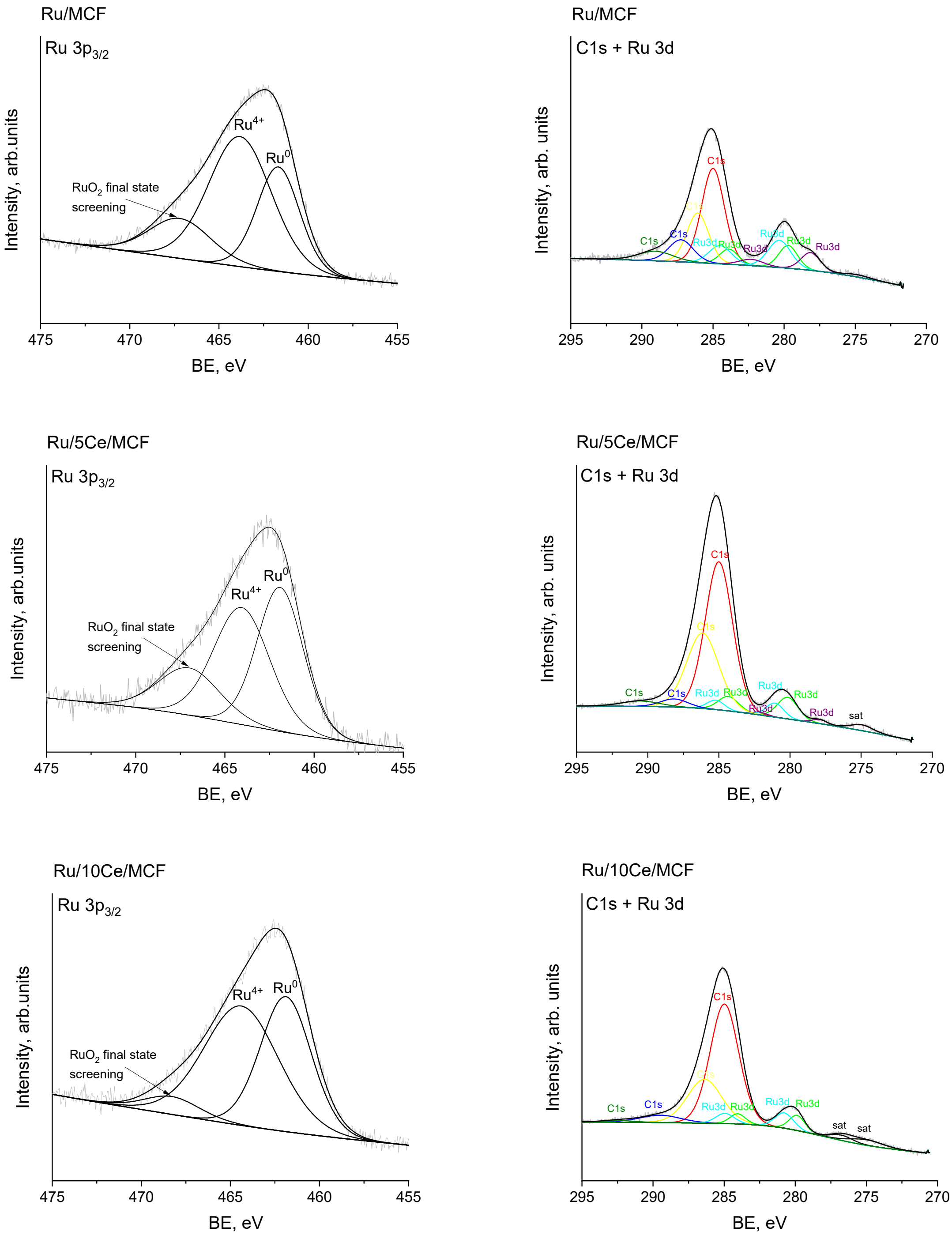

Ru/MCF
Ru 3p3/2
Intensity, arb.units
Ru4+
Ru0
RuO2 final state screening
475 470 465 460 455
BE, eV
Ru/MCF
C1s + Ru 3d
Intensity, arb. units
C1s
Ru3d
295 290 285 280 275 270
BE, eV
Ru/5Ce/MCF
Ru 3p3/2
Intensity, arb.units
Ru4+
Ru0
RuO2 final state screening
475 470 465 460 455
BE, eV
Ru/5Ce/MCF
C1s + Ru 3d
Intensity, arb. units
C1s
Ru3d
sat
295 290 285 280 275 270
BE, eV
Ru/10Ce/MCF
Ru 3p3/2
Intensity, arb.units
Ru4+
Ru0
RuO2 final state screening
475 470 465 460 455
BE, eV
Ru/10Ce/MCF
C1s + Ru 3d
Intensity, arb. units
C1s
Ru3d
sat
295 290 285 280 275 270
BE, eV

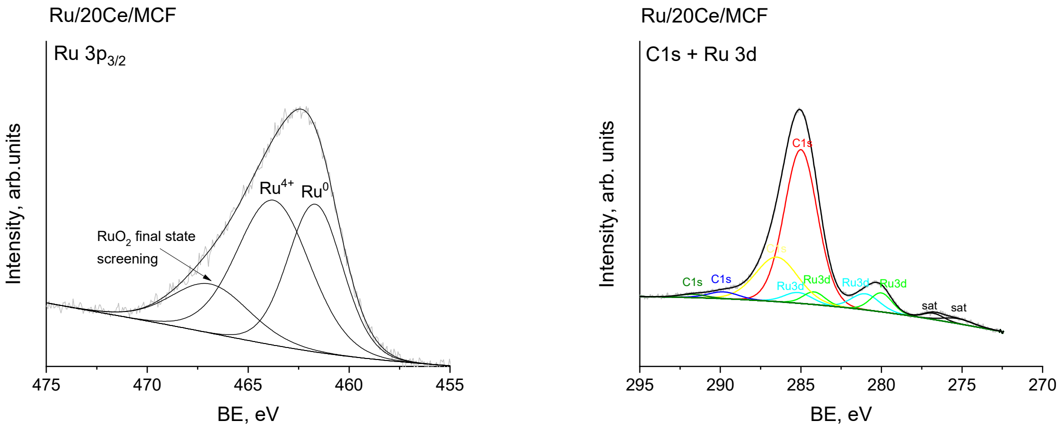

Figure S2. XP spectra in Ru $3p_{3/2}$ and Ru 3d region.
